# Rapid Identification of Chemoresistance Mechanisms Using Yeast DNA Mismatch Repair Mutants

**DOI:** 10.1534/g3.115.020560

**Published:** 2015-07-21

**Authors:** Irene Ojini, Alison Gammie

**Affiliations:** Department of Molecular Biology, Princeton University, Princeton, New Jersey 08544

**Keywords:** drug resistance, DNA mismatch repair, mutator, whole genome sequencing, cancer

## Abstract

Resistance to cancer therapy is a major obstacle in the long-term treatment of cancer. A greater understanding of drug resistance mechanisms will ultimately lead to the development of effective therapeutic strategies to prevent resistance from occurring. Here, we exploit the mutator phenotype of mismatch repair defective yeast cells combined with whole genome sequencing to identify drug resistance mutations in key pathways involved in the development of chemoresistance. The utility of this approach was demonstrated via the identification of the known *CAN1* and *TOP1* resistance targets for two compounds, canavanine and camptothecin, respectively. We have also experimentally validated the plasma membrane transporter *HNM1* as the primary drug resistance target of mechlorethamine. Furthermore, the sequencing of mitoxantrone-resistant strains identified inactivating mutations within *IPT1*, a gene encoding inositolphosphotransferase, an enzyme involved in sphingolipid biosynthesis. In the case of bactobolin, a promising anticancer drug, the endocytosis pathway was identified as the drug resistance target responsible for conferring resistance. Finally, we show that that rapamycin, an mTOR inhibitor previously shown to alter the fitness of the *ipt1* mutant, can effectively prevent the formation of mitoxantrone resistance. The rapid and robust nature of these techniques, using *Saccharomyces cerevisiae* as a model organism, should accelerate the identification of drug resistance targets and guide the development of novel therapeutic combination strategies to prevent the development of chemoresistance in various cancers.

As the global burden of cancer increases, chemoresistance continues to obstruct progress in the long-term control of this disease (Gatti and Zunino 2005). Although chemotherapy is often effective in the short-term, it frequently serves as a potent selective pressure for the proliferation of preexisting resistant variants (Cunningham *et al.* 2011). Furthermore, the heterogeneous nature of most cancers results in an increase in the diversity of resistant phenotypes, thereby increasing the likelihood of therapeutic failure (Nowell 1976). Clinical studies show that most patients will experience a drug resistance–associated relapse within the first few years of initial treatment, resulting in the emergence of an even more aggressive cancer phenotype that is more likely to spread, leading to poor clinical outcomes and survival rates (Agarwal and Kaye 2003; Aguirre-Ghiso 2007; Baniel *et al.* 1995; Engelman and Settleman 2008). Therefore, the identification of drug resistance targets *in vivo* represents an important challenge of significant current interest. Likewise, understanding the genetic basis of chemoresistance can have a direct impact on the development of novel combination therapies.

Previous efforts toward the identification of genes that confer resistance to anticancer drugs have focused on the use of genome-wide screens in *Saccharomyces cerevisiae*, a model eukaryotic organism with considerable homology to the human system (Aouida *et al.* 2004; Burger *et al.* 2000; Furuchi *et al.* 2004; Huang *et al.* 2005; Schenk *et al.* 2003; Wu *et al.* 2004). However, these approaches that rely on high-throughput screening methodologies primarily utilizing the yeast deletion collection or plasmid-based genomic libraries are limited in terms of the constrained range of genetic perturbations explored and the ability to rigorously validate the findings .

In an effort to address these challenges, we developed a rapid and systematic method that exploits the mutator phenotype of mismatch repair defective yeast cells combined with whole genome sequencing to identify mutations that confer resistance to anticancer drugs. Because loss of mismatch repair can increase the mutation rate by a factor of 10^4^, the significant increase in the genetic heterogeneity of the cell population facilitates the selection of drug-resistant variants (Kunkel and Erie 2005; Schofield and Hsieh 2003). The mutation spectrum of mismatch repair defective cells is such that most events are likely to be easily validated inactivating frameshifts within repeat regions (Lang *et al.* 2013; Surtees *et al.* 2004). Additionally, unlike the standard high-throughput methods discussed above, our system allows for the identification of rare mutations within essential genes, thereby expanding the range of resistance mutational events that may be identified. Finally, the use of replicates allows for rigorous validation of the identified resistance target.

Importantly, the experimental conditions used in our analysis are such that the yeast metabolic state resembles rapidly proliferating cancer cells, including increased levels of glucose uptake and fermentation (Warburg effect) as well as changes in amino acid and nucleotide metabolism (Tosato *et al.* 2012; Zimmermann *et al.* 2003). Additionally, the acidity of the yeast medium results in a lower extracellular pH similar to the environment of tumors (Stubbs *et al.* 2003; Stubbs *et al.* 2000).

The identification and evaluation of novel drug combinations to overcome or prevent resistance *in vivo* is another major challenge in cancer drug discovery. As such, we posit that yeast can serve as a powerful model organism for determining whether the pharmacological inhibition of drug-resistant mutant cells will prevent the development of chemoresistance.

In this work, we describe a simple platform capable of rapidly validating previously characterized drug resistance targets and identifying novel genes and pathways responsible for conferring resistance to clinically relevant anticancer compounds. We also provide results demonstrating the use of yeast as a platform for the development of combination therapeutic strategies designed to prevent the evolution of anticancer drug resistance.

## Materials and Methods

### Reagents

A total of 2697 compounds were provided by the National Institutes of Health National Cancer Institute Developmental Therapeutics Program (NCI/DTP) Open Chemical Repository (http://dtp.cancer.gov). The four compound sets screened include: the “Approved Oncology Drugs Set IV” (plates 4762–4763) which consisted of 101 compounds; the “Diversity Set” (plates 4770–4789), which consisted of 1597 compounds; the “Mechanistic Set” (plates 4742–4752), which consisted of 879 compounds; and the “Natural Products Set II” (plates 13120880 and 13120881), which consisted of 120 compounds. The clinically relevant anticancer compounds camptothecin (NSC 94600), mechlorethamine (NSC 762), and bactobolin (NSC 325014) were obtained from the NCI/DTP Open Chemical Repository and selected for further study. Mitoxantrone, rapamycin, FK-506, and etoposide were purchased from Cayman Chemical. Canavanine and DMSO were purchased from Sigma Aldrich. Each compound was dissolved in DMSO.

### Microbial and molecular techniques

Microbial and molecular techniques were described previously (Burke *et al.* 2000). The wild-type strain MY12377 (*his3-11,15 ura3-1 leu2-3,112 RAD5CAN1hom3-10 pdr5∆*::*kanMX erg6∆*::*LEU2*) and *msh2*Δ strain MY12378 (*his3,11,15 ura3-1 leu2-3,112 RAD5CAN1hom3-10 pdr5∆*::*KanMX erg6∆*::*LEU2msh2∆*::*URA3*) are in the W303 strain background but have the wild-type *RAD5* gene.

### Small molecule screens and identification of resistance compounds

The *erg6*∆ *pdr5*∆ knockout strain and an *erg6*∆ *pdr5*∆ *msh2*∆ triple knockout strains described above were grown in synthetic complete media at 30° overnight to reach saturation. The cultures were diluted 1:200 in synthetic complete media and dispensed in a 96-well flat-bottomed microtiter plates with wells containing either the chemical compounds or DMSO as the control. For the NCI/DTP compound libraries, two rounds of screening were performed. In the first round, compounds from the Diversity, Approved Oncology Drug, and Mechanistic sets were screened at a final concentration of 10 μM, whereas compounds from the Natural Product set were screened at a final concentration of 100 μM. Five optical density readings at 600 nm were taken over a 48-hr period using a Perkin Elmer Envision Microplate Reader. Promising compounds were selected for a secondary screen in which optical density readings at 600 nm were taken every 15 min over 48 hr using a BioTek Synergy ^1^H Microplate Reader. The growth rates and lag phases were determined using an R-based program written by Danielle Carpenter and modified by Matthew Cahn (Princeton, NJ).

Compounds that caused a prolonged lag phase in the mutator strain were categorized as resistance due to selection. A prolonged lag phase is defined as approximately two-times the lag phase of the control (DMSO) over 24 hr. Compounds that did not affect the lag phase in the mutator strain were categorized as resistance due to the mismatch repair defective phenotype (Table S2). From this initial screen, resistance hits were defined as compounds that exhibited a 2.5-fold lower OD_600_ reading in *erg6*∆ *pdr5*∆ knockout strain (WT) when compared to the *msh2*∆ *erg6*∆ *pdr5*∆ (*msh2*∆) (*i.e.*, WT OD_600_/*msh2*Δ OD_600_ ≤0.4). The resistance hits were rescreened using a Biotek microplate reader. In this case, selection resistance hits were defined as compounds that exhibited a prolonged lag phase phenotype in *msh2*∆ *erg6*∆ *pdr5*∆ cells (≥24 hr), and condition of mismatch repair deficiency resistance hits were defined as compounds that exhibited a “normal” lag phase phenotype (<24 hr). In the initial NIH screen, two compounds (Celastrol and NSC 1011) causing particularly prolonged lag phases were found (Table S1) and two compounds were categorized as resistance due to the mismatch repair defective phenotype (Table S2).

A second screen was conducted to determine whether more clinically relevant compounds would also cause a prolonged lag phase phenotype at a higher dose. The Approved Oncology Drugs Set IV and the Natural Products Set II compounds were rescreened using the higher-resolution Biotek microplate reader. The metrics for selecting the compounds were as described above. Three compounds in the second screen were categorized as resistance due to selection (Table S1) and one compound was categorized as resistance due to the mismatch repair defective phenotype (Table S2).

In an effort to find more hits, a third screen was conducted with purchased clinically relevant compounds. Dose response analyses were conducted and the resistance phenotype was based on a qualitative assessment of the length of the lag phase as described above. A total of 13 compounds from this screen were categorized as resistance due to selection (Table S1). Six compounds were categorized as resistance due to the mismatch repair defective phenotype (Table S2). The data for all of the screens are found in the Supporting Information (File S1, File S2, File S3, File S4, File S5, File S6).

### Drug resistance discovery platform

Select molecules were chosen to be used in the drug resistance discovery platform described in this article. Camptothecin was chosen as a control because the mode of resistance is known in humans and yeast. Mitoxantrone and mechlorethamine were chosen because they are anticancer drugs currently used in the clinic. Bactobolin was chosen because we found that this molecule specifically inhibited the growth of mismatch repair defective cells. Dose response experiments were conducted for these compounds to determine the optimal concentrations giving rise to resistance in the *erg6*∆ *pdr5*∆ *msh2*∆ triple knockout strain mutator strain. The following range of concentrations were used: canavanine (100 μM–1 mM); camptothecin (10 μM–100 μM); mitoxantrone (10 μM–100 μM); mechlorethamine (10 μM–100 μM); and bactobolin (10 μM–100 μM).

For the canavanine, camptothecin, mechlorethamine, and mitoxantrone resistance experiments, the *msh2*Δ *erg6*∆ *pdr5*∆ strain was inoculated in 5 ml of synthetic complete medium and grown to saturation overnight at 30°. Overnight cultures were diluted 1:200 in synthetic complete media. The diluted *msh2*∆ *erg6*∆ *pdr5*∆ cultures were grown in the presence of the compound at the dose of resistance in 96-well flat-bottomed microtiter plates in a shaking incubator at 30° until saturation was achieved (∼72 hr). Resistance cells were diluted 1:200 in synthetic complete media and dispensed into 96-well flat-bottomed microtiter plates and allowed to grow without drugs in a shaking incubator at 30° for 48 hr. The cultures were then diluted 1:200 in synthetic complete media containing the drug in 96-well flat-bottomed microtiter plates and optical density readings at 600 nm were taken at 15 min intervals over a 48-hr period on a BioTek Synergy ^1^H Microplate Reader. Six independent cultures exhibiting a normal lag phase in the presence of drug were chosen for whole genome sequencing.

For the bactobolin resistance experiments, the *msh2*Δ *erg6*∆ *pdr5*∆ knockout strain was streaked out on nutrient agar plates to allow for selection of single colonies after growth for 2 d at 30°. Ninety-six colonies were selected and each colony was added to an individual well of a microtiter dish containing 200 μl of synthetic complete media. The samples were grown to saturation overnight at 30°. Overnight cultures were diluted 1:200 in synthetic complete media dispensed in a 96-well flat-bottomed microtiter plate. The diluted *msh2*Δ *erg6*∆ *pdr5*∆ cultures were grown in the presence of the compound at the dose conferring resistance in a shaking incubator at 30° for 72 hr. The remainder of the bactobolin experiment was conducted as described above.

### Illumina sequencing and analysis

Samples were prepared for sequencing by inoculating 10 μl from each of the six independent cultures into 20 ml of synthetic complete media. The cells were grown to saturation for 24–48 hr at 30°. Genomic DNA preparations from yeast were performed as previously described (Burke *et al.* 2000). Yeast genomic DNA was prepared for sequencing using the Illumina TruSeq DNA sample preparation kit with indices for multiplexing. Whole genome sequencing was performed at the Lewis-Sigler Institute for Integrative Genomics Core Sequencing Facility using an Illumina HiSequation 2000. Lanes with 9–12 samples each were used. Single end reads of 74 bp were performed, giving an average of 66× coverage of each genome. The sequencing data are available through NCBI (SRA Study Accession Number SRP057759).

The sequence reads were mapped to the draft W303 genome as described previously (Lang *et al.* 2013). Mutations were identified using Freebayes version 0.8.9.a for read groups (Garrison and Marth 2012) using parameters published previously (Lang *et al.* 2013). The chromosomal position of called mutations were processed using YeastMine Genomic DNA Search function (Balakrishnan *et al.* 2012) to determine whether the mutations fell within genes and whether the same gene was mutated in all six isolates.

### Combination therapy studies

The gene-drug homozygous knockout data for *IPT1* deletion strains were acquired from the yeast fitness database *(*http://fitdb.stanford.edu*/)*. Seven clinically relevant compounds shown to cause fitness defects in the *IPT1* deletion strain include: bleomycin; floxuridine; mitomycin C; camptothecin; mechlorethamine; FK-506; and rapamycin. Overnight *msh2*Δ *erg6*∆ *pdr5*∆ cultures were diluted 1:200 in synthetic complete media and added to 96 well plates containing 50 μM mitoxantrone in the absence or presence of the seven compounds: bleomycin (100 nM); mechlorethamine (100 nM); floxuridine (100 μM); mitomycin C (100 μM); FK-506 (100 μM); and rapamycin (1 nM). The compounds were used at a dose that does not inhibit the growth of the *erg6*∆ *pdr5*∆ or *msh2*Δ *erg6*∆ *pdr5*∆ cells. Cultures were grown at 30° with shaking and optical density readings at 600 nm were taken using a Perkin Elmer Envision Microplate Reader. Sample size for each experiment was 40. The error calculation was the SE of proportion.

### Data availability

Strains are available upon request. File S1, File S2, File S3, File S4, File S5, and File S6 contain the raw data from all of the screens. The sequencing data are available through National Center for Biotechnology Information (NCBI) Sequence Read Archive (SRA) Study Accession Number SRP057759.

## Results

### Differences in lag phase distinguish the nature of drug resistance in a mutator strain

**Figure 1:**
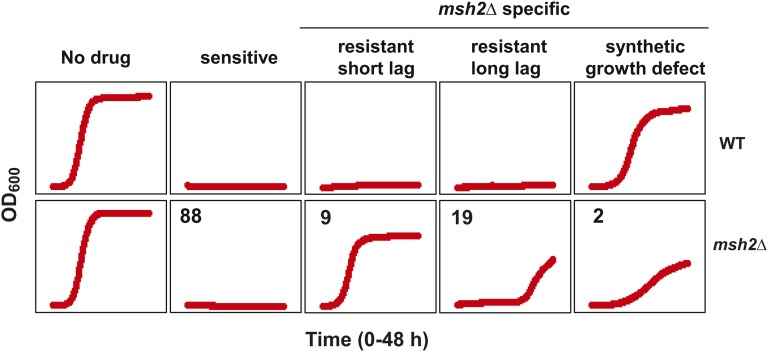
Examples of the effects of compounds of interest from the small molecule screening. Representative growth curves of *erg6*∆ *pdr5*∆ wild-type (WT) and *msh2*Δ *erg6*∆ *pdr5*∆ (*msh2*Δ) strains in the absence of a drug (DMSO control, No Drug), in the presence of a drug that inhibited the growth of both strains (sensitive), in the presence of 400 μM etoposide (resistant, short lag) or 100 μM camptothecin (resistant, long lag), or in the presence of 60 μM bactobolin (synthetic growth defect). Optical density readings at 600 nm (OD_600_) were taken every 15 min for 48 hr using a Biotek Plate Reader. The number of compounds remaining in each category after the screening process is indicated in the upper left of each panel of the *msh2*Δ curves. The three rightmost panels are representative of molecules resulting in an *msh2*∆-specific growth difference.

To identify drugs with high susceptibility to resistance formation, we screened compounds provided by the National Institutes of Health National Cancer Institute Developmental Therapeutics Program (NCI/DTP) Open Chemical Repository. Because yeast cells are often refractory to the effects of small molecules due to the presence of multidrug transporters (encoded by the pleiotropic drug resistance, *PDR*, gene family) and the abundance of ergosterol (*ERG*) in the yeast plasma membrane, an *erg6*∆ *pdr5*∆ knockout strain and an *erg6*∆ *pdr5*∆ *msh2*∆ triple knockout strain were constructed allowing for enhanced sensitivity to small molecules. The compounds were characterized according to their ability to inhibit growth in the mismatch repair proficient strain, the mismatch repair deficient strain or both. Figure 1 summarizes the results of the screening. The leftmost panel shows that in the absence of drug (the DMSO control), there is no difference between the two strains in terms of lag phase, growth rate, or saturation point. The panel second from the left is representative of the 88 compounds that inhibited growth, irrespective of the mismatch repair status (sensitive). The three rightmost panels are representative of molecules resulting in an *msh2*∆-specific growth difference. One of the two compounds causing the *msh2*∆ specific synthetic growth defect is shown in the rightmost panel.

Two types of resistance phenotypes were distinguishable by quantifying the lag phase (Figure 1). For the first resistance phenotype, the mismatch repair defective cultures displayed no significant lag phase, consistent with the resistance being a consequence of the mismatch repair defect (Figure 1, middle panel). The second phenotype, characterized by mismatch repair defective cultures with a long lag phase, was consistent with being caused by mutational events in resistance genes. As mentioned above, mismatch repair deficient cells accumulate mutations at a high rate. If a subpopulation of cells in the starting culture encodes a mutation conferring drug resistance, these cells will eventually dominate the culture when grown in the presence of the drug. Because only a small fraction of the cells in the starting culture carry the drug resistance mutation, it takes a longer time to enter the exponential phase, resulting in a “prolonged lag phase” phenotype, whereas if all the cells in starting culture are resistant, then there will be no significant lag phase. A total of 19 compounds resulted in a prolonged lag phase phenotype (Table S2), and a total of nine compounds resulted in a normal lag phase phenotype (Table 2).

We confirmed that the lag phase could be used to distinguish between the types of resistance by examining the growth curves in response to two control drugs, etoposide, associated with resistance conferred by loss of mismatch repair activity (Aebi *et al.* 1997; de las Alas *et al.* 1997; Fedier *et al.* 2001; Fink *et al.* 1998; Lage and Dietel 1999), and camptothecin, whereby the resistance is caused by a mutational event in the *TOP1* locus (Knab *et al.* 1993; Pommier 2006). The middle panel in Figure 1 shows the normal lag phase for the etoposide-treated cells compared to the prolonged lag phase for the camptothecin treated cultures (second from the right).

### Whole genome sequencing of drug-resistant isolates is an effective method for identifying resistance targets

Exploiting the ability of mutator strains to generate mutations conferring resistance to anticancer drugs at a high rate, we developed a simple platform described below for the rapid identification of genes involved in the development of chemoresistance. We used chemically sensitive mismatch repair null cells to allow for the rapid selection of resistant variants combined with whole genome sequencing methods to identify the genetic alterations responsible for drug resistance. The method involves the propagation of a drug-sensitized mutator strain in microtiter dish wells containing the drug of interest. Mismatch repair defective cultures displaying resistance based on a prolonged lag phase in the presence of the drug were then passaged through a round of exponential growth in the absence of the drug. Finally, cells were cultured in fresh medium in the presence of the drug and cultures that no longer had a significant lag phase were selected for genomic DNA extraction for whole genome sequencing. The absence of a long lag phase after relieving the selective pressure ensures that the resistance was a consequence of a mutational event and not caused by regulatory mechanisms. As controls for the screening method, we examined two drugs, canavanine and camptothecin, each with known targets of resistance.

**Figure 2:**
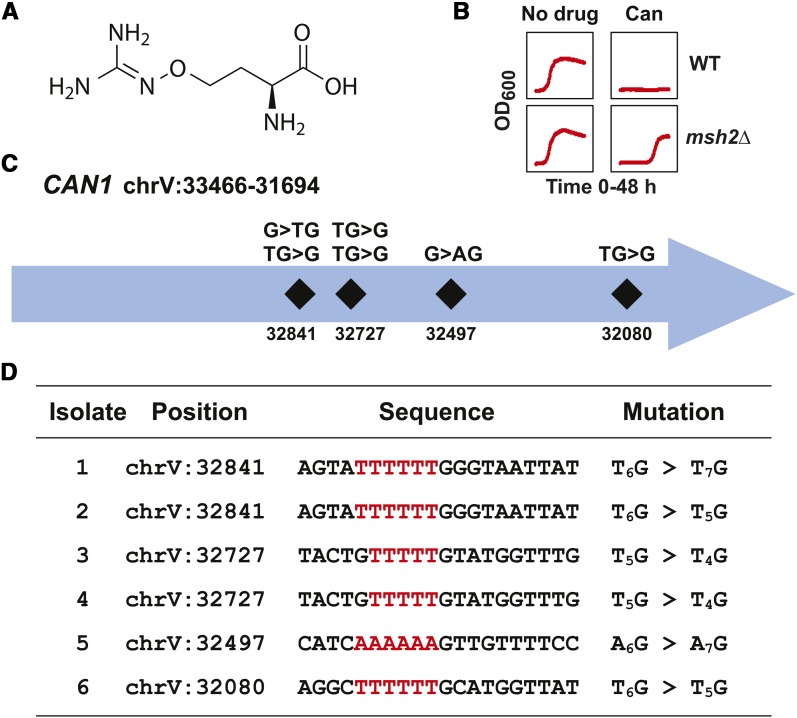
Verification of the resistance discovery method using canavanine. (A) The chemical structure of canavanine. The structure was rendered using ChemDraw. (B) Growth curves of *erg6*∆ *pdr5*∆ wild-type (WT) and *msh2*Δ *erg6*∆ *pdr5*∆ (*msh2*Δ) strains in the absence (No Drug) and presence of 200 μM canavanine (Can). Optical density readings at 600 nm (OD_600_) were taken every 15 min for 48 hr. (C) Schematic representation of the frameshift positions within the *CAN1* locus on chromosome V (chrV) conferring resistance to canavanine. The numbers indicate the chromosomal position. The mutations all resulted in frameshifts at homopolymers detailed in the bottom panel. (D) A table listing the mutations in *CAN1* conferring resistance to canavanine. The nucleotide position for each mutation is shown along with the region mutated. Because *CAN1* is in the opposite orientation (chrV:33466-31694) within the W303 reference genome, the sequence shown is the reverse complement of the reference genome for the given interval. The insertion or deletion at the homopolymeric run (highlighted in red) is indicated for each isolate, for example, a deletion at an A_6_ repeat followed by a C (AAAAAAC) would be designated A_6_C > A_5_C, whereas an insertion would be CA_6_ > CA_7_.

Canavanine (Figure 2A) is a toxic analog of arginine taken into the cells via the arginine permease encoded at the *CAN1* locus (Broach *et al.* 1979; Rosenthal 1977). As mentioned above, if the *CAN1* gene is inactivated, then the yeast become resistant to canavanine (Whelan *et al.* 1979). At a concentration of ∼200 μM, a typical mutator culture will show a long lag phase consistent with mutational event resistance (Figure 2B). To identify genomic changes associated with canavanine resistance, whole genome sequencing of six independently isolated canavanine-resistant cultures was performed. All of the isolates harbored frameshifts within the *CAN1* gene (Figure 2C). The mutations were all single base insertions or deletions within homopolymer repeats (HPR) (Figure 2D). Although two isolates share the same frameshift mutation in *CAN1*, they do not share any other mutations in common, suggesting that these inactivating events occurred independently.

**Figure 3:**
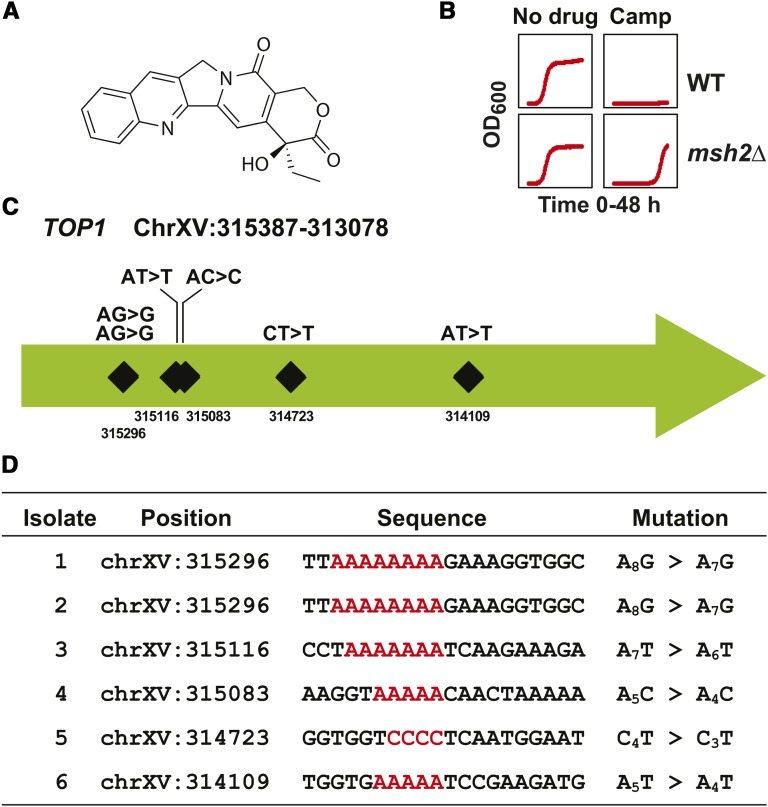
Verification of resistance discovery method using camptothecin. (A) Chemical structure of camptothecin. The structure was rendered using ChemDraw. (B) Growth curves of *erg6*∆ *pdr5*∆ wild-type (WT) and *msh2*Δ *erg6*∆ *pdr5*∆ (*msh2*Δ) strains in the absence (No Drug) and presence of 9 μM camptothecin (Camp). Optical density readings at 600 nm (OD_600_) were taken every 15 min for 48 hr. (C) Schematic representation of the frameshift positions within the *TOP1* locus on chromosome XV (chrXV) conferring resistance to camptothecin. The numbers indicate the chromosomal position. The mutations all resulted in frameshifts at homopolymers detailed in the bottom panel. (D) A table listing the mutations in *TOP1* conferring resistance to camptothecin. The nucleotide position for each mutation is shown along with the region mutated. The sequence given corresponds to the strand in the W303 reference genome. Because *TOP1* is in the opposite orientation (chrXV:315387-313078) within the W303 reference genome, the sequence shown is the reverse complement of the reference genome for the given interval. The nucleotide numbers differ slightly from the S288C draft genome. The specific insertion or deletion at the homopolymeric run (indicated in red) is indicated in a format described in Figure 2.

The anticancer compound camptothecin (Figure 3A) is a well-characterized topoisomerase I inhibitor that targets the *TOP1* gene (Knab *et al.* 1993). Consistent with a mutational event resistance phenotype, the camptothecin resistance is characterized by a long lag phase (Figure 3B). As with canavanine, we identified six independent isolates with mutations in the *TOP1* gene (Figure 3C). All six mutations represented frameshift mutations within homopolymer nucleotide stretches (Figure 3D). Taken together, this method proved to be a simple and rapid way to identify the drug resistance target for compounds when the loss of function of a single locus confers resistance.

### The *Hnm1* transmembrane protein is the major resistance target for mechlorethamine

**Figure 4:**
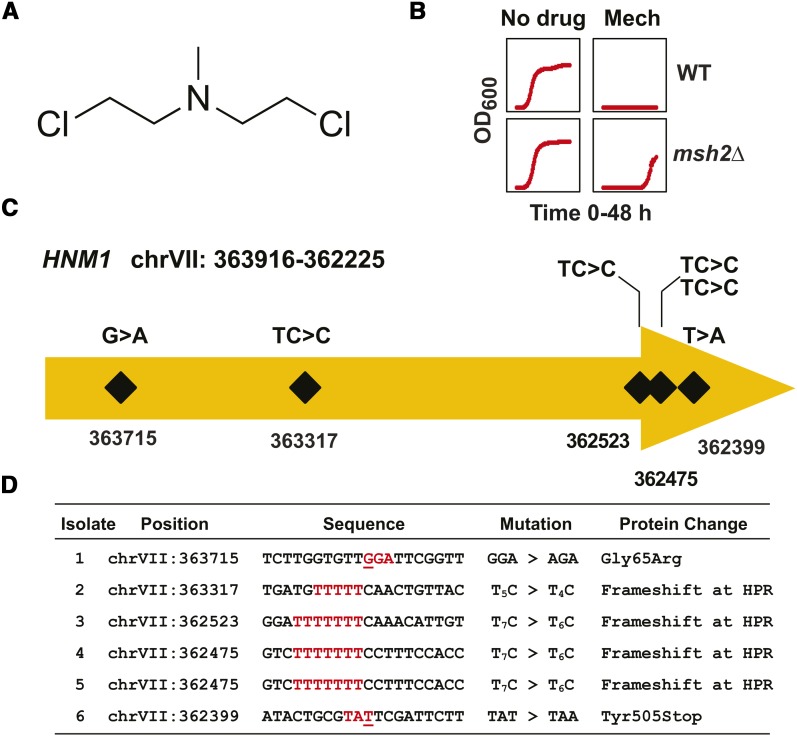
Inactivation of the Hnm1 transporter is the major cause of resistance to mechlorethamine. (A) Chemical structure of mechlorethamine. The structure was rendered using ChemDraw. (B) Growth curves of *erg6*∆ *pdr5*∆ wild-type (WT) and *msh2*Δ *erg6*∆ *pdr5*∆ (*msh2*Δ) strains in the absence (No Drug) and presence of 70 μM mechlorethamine (Mech). Optical density readings at 600 nm (OD_600_) were taken every 15 min for 48 hr. (C) Schematic representation of the frameshift positions within the *HNM1* locus on chromosome VII (chrVII) conferring resistance to mechlorethamine. The numbers indicate the chromosomal position. The mutations were frameshifts at homopolymers, a missense mutation (GGA > AGA), and a nonsense mutation (TAT > TAA), and are detailed in the bottom panel. (D) A table listing the mutations in *HNM1* conferring resistance to mechlorethamine. The nucleotide position for each mutation is shown along with the region mutated. The sequence given corresponds to the strand in the W303 reference genome. Because *HNM1* is in the opposite orientation (chrVII: 363916–362225) within the W303 reference genome, the sequence shown is the reverse complement of the reference genome for the given interval. The nucleotide numbers differ slightly from the S288C draft genome. The mutated nucleotides for the point mutations are underlined. The mutated codons and homopolymers are indicated in red. The specific insertion or deletion at the homopolymeric run is indicated in a format described in Figure 2.

Mechlorethamine (Figure 4A) is a frequently prescribed drug used to treat Hodgkin lymphoma and several other cancers (Gold *et al.* 1970). Mechlorethamine is an alkylating agent and inhibits DNA replication via the formation of interstrand crosslinks (Millard *et al.* 1990; Rink and Hopkins 1995). We found that the growth curves of mechlorethamine resistance are consistent with a mutational event resistance (Figure 4B). Whole genome sequencing of mechlorethamine-resistant strains revealed *HNM1* as the primary resistance locus. *HNM1* encodes a small molecule transporter that presumably allows for entry of mechlorethamine within the cell (Li and Brendel 1993). We identified four frameshifts within homopolymeric repeats, a nonsense mutation generating a truncation, and a missense mutation within a transmembrane region (Figure 4, C and D). The missense mutation was a C-to-T substitution causing the glycine at amino acid 65 to be changed to an arginine (G65R). G65 is located in transmembrane domain I and substitution of the arginine results in the introduction of a positive charge likely to destabilize the hydrophobic transmembrane domain. Taken together, our assay provides direct evidence that loss of function of *HNM1* is the major drug-resistant target for mechlorethamine.

### Mutations in genes in the endocytosis pathway confer resistance to bactobolin

**Figure 5:**
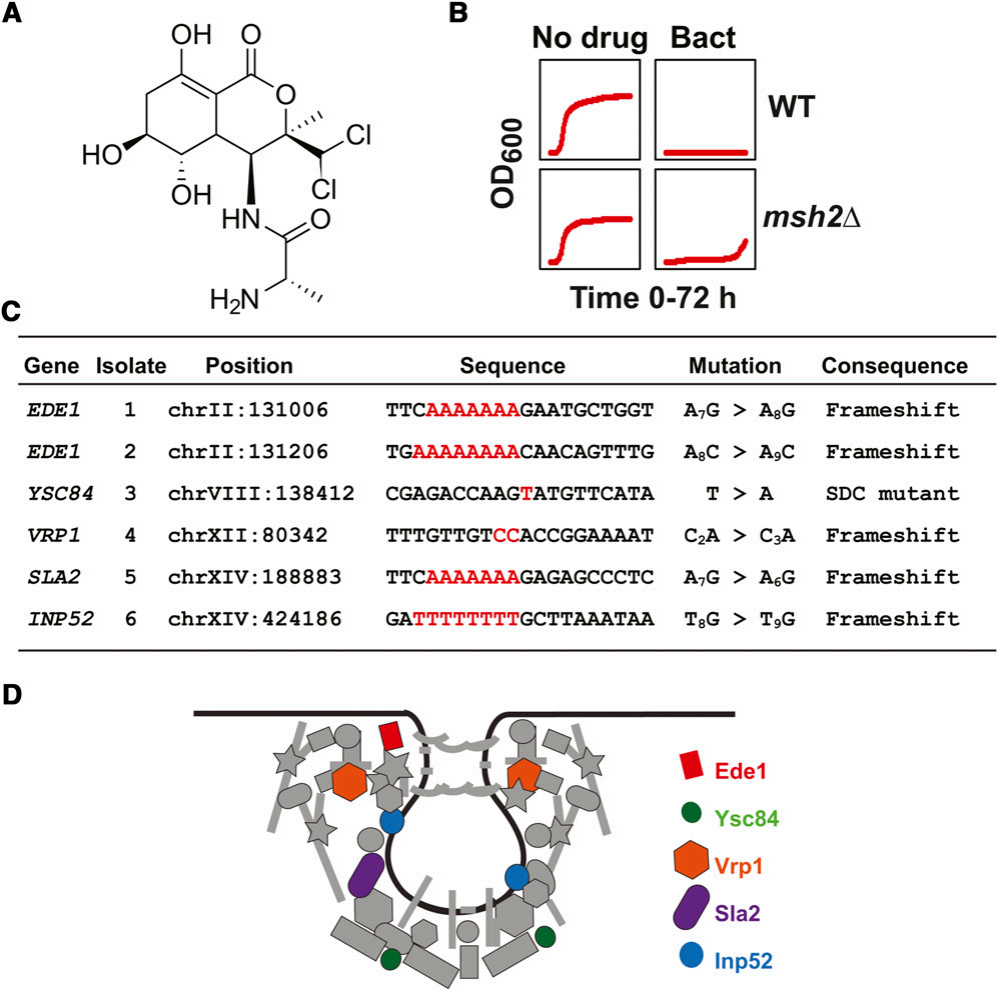
Mutations in the endocytosis pathway confer resistance to bactobolin. (A) Chemical structure of bactobolin. The structure was rendered using ChemDraw. (B) Growth curves of *erg6*∆ *pdr5*∆ wild-type (WT) and *msh2*Δ *erg6*∆ *pdr5*∆ (*msh2*Δ) strains in the absence (No Drug) and presence of 100 μM bactobolin (Bact). Optical density readings at 600 nm (OD_600_) were taken every 15 min for 72 hr. (C) The table lists the genes and mutations conferring resistance to bactobolin. The coding strand nucleotide sequence and mutation for each isolate is shown. Five caused inactivating frameshifts and one (within *YSC84*) resulted in a mutation in the splice donor consensus sequence (SDC). (D) A schematic drawing of the clathrin-dependent endocytic pathway is adapted from a previously published model (Weinberg and Drubin 2012). The black line represents a membrane undergoing endocytosis. The endocytosis components that were identified are represented with colors: Ede1 (red); Ysc84 (green); Vrp1 (orange); Sla2 (purple); and Inp52 (blue). Other endocytosis components are in gray.

Bactobolin (Figure 5A), an anticancer compound with protein synthesis inhibitory activity, has demonstrated *in vitro* cytotoxicity against the human melanoma cell line B16 and has also prolonged the survival time of mice with leukemia L-1210 (Ishizuka *et al.* 1980; Kawada *et al.* 1999). Therefore, the potent anticancer activity of bactobolin necessitates further investigation into possible modes of resistance, allowing for the design of bactobolin-based combination therapies. As with the other compounds used in this analysis, the resistance profile for bactobolin is consistent with a mutational event (Figure 5B). The results from the bactobolin resistance experiments failed to reveal a single resistance locus; however, a gene ontology analysis showed there was a significant enrichment of genes involved in endocytosis. Each isolate contained an inactivating mutation within a gene in the endocytosis pathway; five were frameshift events and one destroyed a splice donor consensus sequence (Figure 5, C and D). Taken together, the results suggest that the endocytosis pathway is important for the entry of bactobolin into the cells. Furthermore, the results show that a pathway is also easily identified by using this simple platform.

### *IPT1*, a gene implicated in sphingolipid biosynthesis, is the major resistance target for cells treated with mitoxantrone

**Figure 6:**
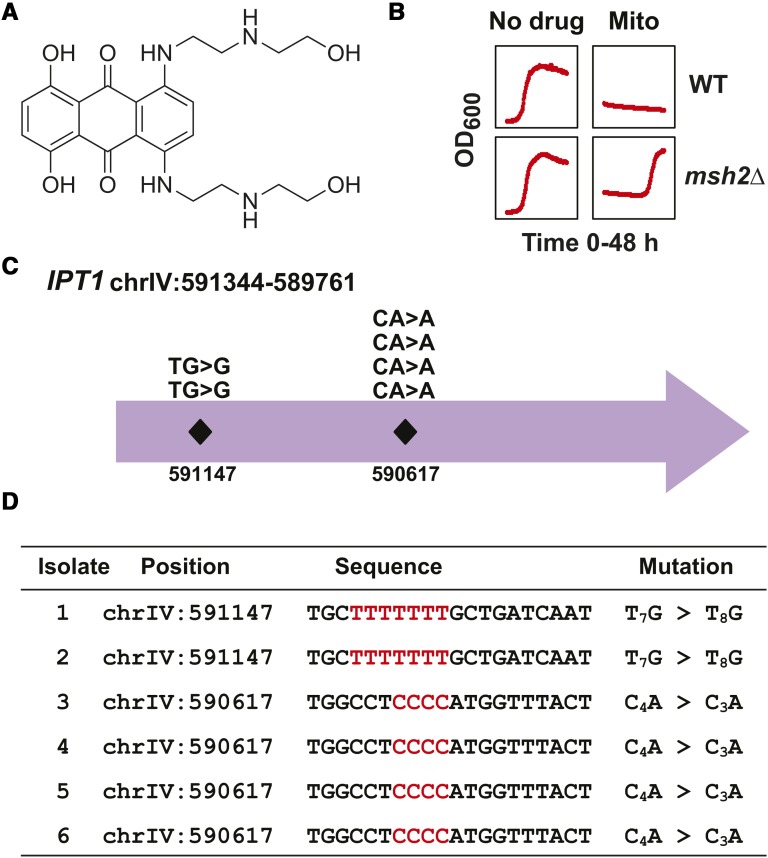
Inactivation of Ipt1 is the major cause of resistance to mitoxantrone. (A) Chemical structure of mitoxantrone. The structure was rendered with ChemDraw. (B) Growth curves of *erg6*∆ *pdr5*∆ wild-type (WT) and *msh2*Δ *erg6*∆ *pdr5*∆ (*msh2*Δ) strains in the absence and presence of mitoxantrone (50 μM). Optical density readings at 600 nm (OD_600_) were taken every 15 min for 48 hr. The OD_600_ offset observed in the presence of the drug is due to the colored nature of mitoxantrone. (C) Schematic representation of the frameshift positions within the *IPT1* locus on chromosome IV (chrIV) conferring resistance to mitoxantrone. The numbers indicate the chromosomal position. The mutations all resulted in frameshifts at homopolymers detailed in the bottom panel. (D) A table listing the mutations in *IPT1* conferring resistance to mitoxantrone. The nucleotide position for each mutation is shown along with the region mutated. The sequence given corresponds to the strand in the W303 reference genome. Because *IPT1* is in the opposite orientation (chrIV: 591344–589761) within the W303 reference genome, the sequence shown is the reverse complement of the reference genome for the given interval. The nucleotide numbers differ slightly from the S288C draft genome. The specific insertion or deletion at the homopolymeric run is indicated in a format described in Figure 2.

Mitoxantrone (Figure 6A) is a commonly prescribed drug used to treat a wide variety of cancers and autoimmune diseases (Hande 1998). Mitoxantrone inhibits rapidly dividing cells by targeting topoisomerase II during DNA synthesis (Bellosillo *et al.* 1998; Fox and Smith 1990). The mitoxantrone resistance phenotype is consistent with a mutational event resistance (Figure 6B). We identified the mutations within six isolates and found that the *IPT1* gene is the major target for mitoxantrone resistance (Figure 6C). All of the isolates had frameshift mutations at homopolymeric repeats within the *IPT1* gene (Figure 6D). *IPT1* encodes an inositolphosphotransferase involved in synthesis of mannose-(inositol-phosphate)_2_-ceramide, with the most abundant sphingolipid in yeast (Dickson *et al.* 1997). These data suggest that entry of mitoxantrone may depend on lipid rafts or essential transporters within lipid rafts. Interestingly, in human cell lines, mitoxantrone resistance has been linked to differences in plasma membrane permeability (Breuzard *et al.* 2005).

### Combination therapeutic approaches are capable of blocking resistance formation

The development of simple and rapid methods for developing effective combination therapies designed to prevent the emergence of drug-resistant variants remains a tremendous challenge in cancer research. Our approach to overcoming this challenge involves the use of the yeast fitness database (FitDB), which is a genome-wide collection of fitness profiles for ∼4800 single gene yeast deletion strains in response to 400 small molecules and a wide variety of different growth conditions (Hillenmeyer *et al.* 2010; Hillenmeyer *et al.* 2008). To narrow the list of clinically used drugs capable of targeting and inhibiting the growth of mitoxantrone-resistant cells, we searched the fitness database for drugs that can cause a fitness defect in cells carrying *IPT1* null mutations, which results in mitoxantrone resistance. The compounds selected for this study include bleomycin, floxuridine, mitomycin C, camptothecin, mechlorethamine, FK-506, and rapamycin. The compounds used in combination with mitoxantrone do not have any obvious phenotype when administered singly to the *msh2erg6pdr5* strain at the low doses used for the combinations. Although all compounds were able to inhibit the development of resistance (Table 1), the combination of mitoxantrone and a TOR inhibitor (rapamycin and FK-506) appear to be the most effective regimens in the prevention of mitoxantrone resistance. This study demonstrates the utility of yeast as a tool for the development of combination therapeutic strategies.

**Table 1:** Drug combinations to prevent mitoxantrone resistance

Drug Combination	Mitoxantrone-Resistant Cultures (n = 40)
Mitoxantrone + DMSO control	98 ± 2%
Mitoxantrone + mechlorethamine	74 ± 7%
Mitoxantrone + floxuridine	64 ± 8%
Mitoxantrone + bleomycin	28 ± 7%
Mitoxantrone + camptothecin	13 ± 5%
Mitoxantrone + mitomycin C	3 ± 3%
Mitoxantrone + rapamycin	0%
Mitoxantrone + FK506 rapamycin analog	0%

Error bars reflect the standard error of proportion.

## Discussion

### Summary

Chemoresistance represents a primary challenge in medical treatments for conditions ranging from cystic fibrosis to cancer (Stefanko and Wrobel 2010). Although chemoresistance continues to be a significant impediment to effective medical treatments, the number of strategies designed to address resistance is limited. The ability to rapidly identify drug resistance mechanisms would provide the information necessary for the development of more effective treatment strategies. In this work, we describe a simple platform that exploits the mutator phenotype of mismatch repair defective yeast cells combined with whole genome sequencing to identify drug resistance mutations in key pathways involved in the development of chemoresistance. Using a mismatch repair defective mutator strain allows for a spectrum of inactivating mutations, including frameshift, nonsense, missense, and splice-site mutations in drug resistance candidate genes. In this work we also outline a method that rapidly screens drugs that target resistant cells to use in combination and prevent resistance formation. Using mitoxantrone resistance as a case study, we showed that this platform provides an efficient method for evaluating and prioritizing effective drug combinations capable of preventing the emergence of resistant cells from dominating a population.

### Using a yeast mutator strain allows for the rapid identification and validation of drug resistance targets with inactivating mutations in single genes, gene pathways, and essential genes

Prior to the completion of the yeast deletion collection, the process for identifying resistance targets involved chemical or ultraviolet light mutagenesis followed by the selection of drug-resistant mutants. After identification of resistant isolates, a thorough genetic characterization of the resistance alleles was required before classical cloning methods and linkage analysis fully verified the genetic basis of the resistance. The methods were thorough, but laborious. The platform described in this work allows for the rapid identification and verification of drug resistance targets and is broadly applicable to any compounds resulting in a resistance phenotype. The preponderance of frameshifts in mismatch repair defective cells allows for a straightforward characterization of the molecular nature of the resistance. Unlike screens using the yeast deletion collection, this method is able to characterize rare alleles of essential genes responsible for conferring resistance.

In this work we showed that frameshift mutations identified in *CAN1* and *TOP1*, drug resistance targets for canavanine and camptothecin, respectively, validated the use of this platform for determining the genetic basis of drug resistance. Identification of a variety of mutation types (nonsense, frameshift, and missense) in the *HNM1* transporter gene for mechlorethamine-resistant cells confirmed the effectiveness of this technique for validating previous findings (Li and Brendel 1993). Additionally, we identified a novel resistance mechanism for mitoxantrone involving sphingolipid biosynthesis. The mitoxantrone-resistant cells all contained frameshift mutations in the sphingolipid biosynthetic gene *IPT1*, suggesting that the primary mechanism of resistance is reduced membrane permeability. Although most of the resistance mechanisms involved single gene events, we also demonstrated that pathways are readily identifiable for certain drug resistance phenotypes; for example, bactobolin resistance was shown to be a consequence of mutations in endocytosis genes.

### The resistance targets are likely to be similar in mammalian cells

An important issue is the transferability of the findings to mammalian cells. In the case of camptothecin, the resistance target in yeast and humans are identical. In both cases, inactivating mutations in topoisomerase 1 render the cell resistant to the cytotoxic effects of camptothecin (Pommier 2006). Additionally, in yeast and humans, mechlorethamine is taken up by the choline transporter (Doppler *et al.* 1988; Lerner 1989; Li and Brendel 1993). Therefore, the mechanism of resistance to mechlorethamine is expected to be similar. In the case of mitoxantrone, amphiphilic drugs like mitoxantrone or its analogs (*e.g.*, doxorubicin) get into the cell by passive diffusion (Veldman *et al.* 2004). Therefore, altering the composition of the plasma membrane via sphingolipid biosynthesis should cause resistance to amphiphilic drugs like mitoxantrone in yeast and humans (Sietsma *et al.* 2001). It is of interest that in human cell lines, mitoxantrone resistance is associated with differences in plasma membrane permeability (Breuzard *et al.* 2005). Finally, a common mechanism of resistance is the reduction of intracellular drug levels that can occur via a variety of ways including inhibiting endocytosis, as in the case of bactobolin (Gottesman 2002). In summary, all of the resistance targets uncovered in this analysis are likely to be applicable to mammalian cells.

### Yeast as an organism for drug discovery for the treatment of human diseases

*Saccharomyces cerevisiae* has a long history in the field of chemical genomics (Baetz *et al.* 2004; Giaever *et al.* 1999, 2004; Hoon *et al.* 2008; Hughes *et al.* 2000; Lum *et al.* 2004; Parsons *et al.* 2004, 2006; Pommier 2006). Its complete and well-annotated genome and highly conserved genetic and biological pathways make it a powerful tool for the mechanistic investigation of clinically relevant bioactive compounds. For instance, rapamycin is an immunosuppressive drug with anticancer properties (Guertin and Sabatini 2007; Vignot *et al.* 2005). The target of rapamycin (TOR) proteins were originally identified via the genetic screening of rapamycin-resistant clones in yeast (Heitman *et al.* 1991; Lorenz and Heitman 1995). These proteins were later found to be functionally conserved from yeast to humans (Cutler *et al.* 1999). Likewise, the discovery of the copper transporter *CTR1* was first discovered in yeast and was later found to be the cause of cisplatin resistance in humans (Lin *et al.* 2002; Zhou and Gitschier 1997). As a final example, a yeast chemical genetic screen identified splitomycin as an inhibitor of the highly conserved Sir2 histone acetyltransferase (Bedalov *et al.* 2001). Splitomycin has since been used for as a mechanistic probe of SIRT1, the human homolog of Sir2 (Westphal *et al.* 2007). In summary, yeast is a powerful model organism with a proven history of facilitating the understanding of the molecular mechanisms of drug action and resistance that are applicable to treating human disease in the clinic.

### The use of yeast to efficiently find combination drug therapies to prevent chemoresistance formation in patients

Once the mechanism of resistance has been determined, combination therapies could potentially allow for the direct targeting of resistant mechanisms. Eradication of resistant populations would significantly reduce the commonplace problem of cancer recurrence. The experimental convenience of *Saccharomyces cerevisiae* as a model organism allows for the screening of potential drugs capable of targeting resistance mechanisms. Our results suggest that the combination of an anticancer drug and a drug known to reduce the fitness of cells carrying null mutations in genes conferring resistance can be an effective strategy for tackling chemoresistance. We quantified the effectiveness of dual treatments and found that the combination of mitoxantrone and rapamycin completely prevented the emergence of mitoxantrone-resistant cells. Evidence for the ability of TOR inhibitors to prevent and reverse chemoresistance has been documented in human cells with mitoxantrone analogs, doxorubicin, and daunomycin (Arceci *et al.* 1992; Grunwald *et al.* 2002; Mondesire *et al.* 2004). In conclusion, this report describes a rapid method for identifying drug resistance mutations and identifying cotreatment strategies that prevent the selection and evolution of resistance phenotypes. The speed and accuracy of our approach demonstrates the potential applications of this technique.

## Supplementary Material

Supporting Information

## References

[bib1] Aebi S. Fink D. Gordon R. Kim H. K. Zheng H., 1997 Resistance to cytotoxic drugs in DNA mismatch repair-deficient cells. Clin. Cancer Res. 3: 1763–1767. 9815561

[bib2] Agarwal R. Kaye S. B., 2003 Ovarian cancer: strategies for overcoming resistance to chemotherapy. Nat. Rev. Cancer 3: 502–516. 12835670 10.1038/nrc1123

[bib3] Aguirre-Ghiso J. A., 2007 Models, mechanisms and clinical evidence for cancer dormancy. Nat. Rev. Cancer 7: 834–846. 17957189 10.1038/nrc2256 PMC2519109

[bib4] Aouida M. Page N. Leduc A. Peter M. Ramotar D., 2004 A genome-wide screen in Saccharomyces cerevisiae reveals altered transport as a mechanism of resistance to the anticancer drug bleomycin. Cancer Res. 64: 1102–1109. 14871844 10.1158/0008-5472.can-03-2729

[bib5] Arceci R. J. Stieglitz K. Bierer B. E., 1992 Immunosuppressants FK506 and rapamycin function as reversal agents of the multidrug resistance phenotype. Blood 80: 1528–1536. 1381629

[bib6] Baetz K. McHardy L. Gable K. Tarling T. Reberioux D., 2004 Yeast genome-wide drug-induced haploinsufficiency screen to determine drug mode of action. Proc. Natl. Acad. Sci. USA 101: 4525–4530. 15070751 10.1073/pnas.0307122101 PMC384780

[bib7] Balakrishnan, R., J. Park, K. Karra, B. C. Hitz, G. Binkley *et al.*, 2012 YeastMine-an integrated data warehouse for Saccharomyces cerevisiae data as a multipurpose tool-kit. Database (Oxford). 2012: bar062. 10.1093/database/bar062 PMC3308152 22434830

[bib8] Baniel J. Foster R. S. Gonin R. Messemer J. E. Donohue J. P., 1995 Late relapse of testicular cancer. J. Clin. Oncol. 13: 1170–1176. 7537800 10.1200/JCO.1995.13.5.1170

[bib9] Bedalov A. Gatbonton T. Irvine W. P. Gottschling D. E. Simon J. A., 2001 Identification of a small molecule inhibitor of Sir2p. Proc. Natl. Acad. Sci. USA 98: 15113–15118. 11752457 10.1073/pnas.261574398 PMC64992

[bib10] Bellosillo B. Colomer D. Pons G. Gil J., 1998 Mitoxantrone, a topoisomerase II inhibitor, induces apoptosis of B-chronic lymphocytic leukaemia cells. Br. J. Haematol. 100: 142–146. 9450803 10.1046/j.1365-2141.1998.00520.x

[bib11] Breuzard G. Piot O. Angiboust J. F. Manfait M. Candeil L., 2005 Changes in adsorption and permeability of mitoxantrone on plasma membrane of BCRP/MXR resistant cells. Biochem. Biophys. Res. Commun. 329: 64–70. 15721274 10.1016/j.bbrc.2005.01.098

[bib12] Broach J. R. Strathern J. N. Hicks J. B., 1979 Transformation in yeast - development of a hybrid cloning vector and isolation of the *CAN1* gene. Gene 8: 121–133. 395029 10.1016/0378-1119(79)90012-x

[bib13] Burger H. Capello A. Schenk P. W. Stoter G. Brouwer J., 2000 A genome-wide screening in Saccharomyces cerevisiae for genes that confer resistance to the anticancer agent cisplatin. Biochem. Biophys. Res. Commun. 269: 767–774. 10720490 10.1006/bbrc.2000.2361

[bib14] Burke D. Dawson D. Stearns T., and Cold Spring Harbor Laboratory, 2000 *Methods in yeast genetics: a Cold Spring Harbor Laboratory course manual*. Cold Spring Harbor Laboratory Press, Plainview, NY.

[bib15] Cunningham J. J. Gatenby R. A. Brown J. S., 2011 Evolutionary dynamics in cancer therapy. Mol. Pharm. 8: 2094–2100. 21815657 10.1021/mp2002279 PMC3250072

[bib16] Cutler N. S. Heitman J. Cardenas M. E., 1999 TOR kinase homologs function in a signal transduction pathway that is conserved from yeast to mammals. Mol. Cell. Endocrinol. 155: 135–142. 10580846 10.1016/s0303-7207(99)00121-5

[bib17] de las Alas, M. M., S. Aebi, D. Fink, S. B. Howell and G. Los, 1997 Loss of DNA mismatch repair: Effects on the rate of mutation to drug resistance. J Natl Cancer Inst. 89: 1537–1541. 10.1093/jnci/89.20.1537 9337351

[bib18] Dickson R. C. Nagiec E. E. Wells G. B. Nagiec M. M. Lester R. L., 1997 Synthesis of mannose-(inositol-P)(2)-ceramide, the major sphingolipid in Saccharomyces cerevisiae, requires the IPT1 (YDR072c) gene. J. Biol. Chem. 272: 29620–29625. 9368028 10.1074/jbc.272.47.29620

[bib19] Doppler W. Hofmann J. Maly K. Grunicke H. H., 1988 Protection of Ehrlich ascites tumor-cells against the antiproliferative effect of mechlorethamine (nitrogen-mustard) by 5-N,N-dimethylamiloride. Cancer Res. 48: 2454–2457. 3356009

[bib20] Engelman J. A. Settleman J., 2008 Acquired resistance to tyrosine kinase inhibitors during cancer therapy. Curr. Opin. Genet. Dev. 18: 73–79. 18325754 10.1016/j.gde.2008.01.004

[bib21] Fedier A. Schwarz V. A. Walt H. Carpini R. D. Haller U., 2001 Resistance to topoisomerase poisons due to loss of DNA mismatch repair. Int. J. Cancer 93: 571–576. 11477562 10.1002/ijc.1356

[bib22] Fink D. Aebi S. Howell S. B., 1998 The role of DNA mismatch repair in drug resistance. Clin. Cancer Res. 4: 1–6. 9516945

[bib23] Fox M. E. Smith P. J., 1990 Long-term inhibition of DNA-synthesis and the persistence of trapped topoisomerase-Ii complexes in determining the toxicity of the antitumor DNA intercalators messenger Amsa and mitoxantrone. Cancer Res. 50: 5813–5818. 2168281

[bib24] Furuchi T. Nitta K. Takahashi T. Naganuma A., 2004 Overexpression of Ssl2p confers resistance to adriamycin and actinomycin D in Saccharomyces cerevisiae. Biochem. Biophys. Res. Commun. 314: 844–848. 14741713 10.1016/j.bbrc.2003.12.160

[bib25] Garrison, E., and G. Marth, 2012 Haplotype-based variant detection from short-read sequencing. arXiv preprint arXiv:1207.3907.

[bib26] Gatti L. Zunino F., 2005 Overview of tumor cell chemoresistance mechanisms. Methods Mol. Med. **111:** 127–148. 10.1385/1-59259-889-7:127 15911977

[bib27] Giaever G. Shoemaker D. D. Jones T. W. Liang H. Winzeler E. A., 1999 Genomic profiling of drug sensitivities via induced haploinsufficiency. Nat. Genet. 21: 278–283. 10080179 10.1038/6791

[bib28] Giaever G. Flaherty P. Kumm J. Proctor M. Nislow C., 2004 Chemogenomic profiling: Identifying the functional interactions of small molecules in yeast. Proc. Natl. Acad. Sci. USA 101: 793–798. 14718668 10.1073/pnas.0307490100 PMC321760

[bib29] Gold G. L. Salvin L. G., M. A. Schneide, J. Colsky, A. H. Owens *et al*, 1970 The use of mechlorethamine, cyclophosphamide, and uracil mustard in neoplastic disease: a cooperative study. J. Clin. Pharmacol. New Drugs 10: 110. 10.1177/009127007001000206 4906539

[bib30] Gottesman M. M., 2002 Mechanisms of cancer drug resistance. Annu. Rev. Med. 53: 615–627. 11818492 10.1146/annurev.med.53.082901.103929

[bib31] Grunwald V. DeGraffenried L. Russel D. Friedrichs W. E. Ray R. B., 2002 Inhibitors of mTOR reverse doxorubicin resistance conferred by PTEN status in prostate cancer cells. Cancer Res. 62: 6141–6145. 12414639

[bib32] Guertin D. A. Sabatini D. M., 2007 Defining the role of mTOR in cancer. Cancer Cell 12: 9–22. 17613433 10.1016/j.ccr.2007.05.008

[bib33] Hande K. R., 1998 Clinical applications of anticancer drugs targeted to topoisomerase II. Biochimica Et Biophysica Acta-Gene Structure and Expression 1400: 173–184. 10.1016/s0167-4781(98)00134-1 9748560

[bib34] Heitman J. Movva N. R. Hall M. N., 1991 Targets for cell cycle arrest by the immunosuppressant rapamycin in yeast. Science 253: 905–909. 1715094 10.1126/science.1715094

[bib35] Hillenmeyer M. E. Fung E. Wildenhain J. Pierce S. E. Hoon S., 2008 The chemical genomic portrait of yeast: Uncovering a phenotype for all genes. Science 320: 362–365. 18420932 10.1126/science.1150021 PMC2794835

[bib36] Hillenmeyer M. E. Ericson E. Davis R. W. Nislow C. Koller D., 2010 Method Systematic analysis of genome-wide fitness data in yeast reveals novel gene function and drug action. Genome Biol. 11: R30. 20226027 10.1186/gb-2010-11-3-r30 PMC2864570

[bib37] Hoon S. Smith A. M. Wallace I. M. Suresh S. Miranda M., 2008 An integrated platform of genomic assays reveals small-molecule bioactivities. Nat. Chem. Biol. 4: 498–506. 18622389 10.1038/nchembio.100

[bib38] Huang R. Y. Eddy M. Vujcic M. Kowalski D., 2005 Genome-wide screen identifies genes whose inactivation confer resistance to cisplatin in Saccharomyces cerevisiae. Cancer Res. 65: 5890–5897. 15994967 10.1158/0008-5472.CAN-04-4093

[bib39] Hughes T. R. Marton M. J. Jones A. R. Roberts C. J. Stoughton R., 2000 Functional discovery via a compendium of expression profiles. Cell 102: 109–126. 10929718 10.1016/s0092-8674(00)00015-5

[bib40] Ishizuka M. Fukasawa S. Masuda T. Sato J. Kanbayashi N., 1980 Anti-tumor effect of bactobolin and its influence on mouse immune-system and hematopoietic-cells. J. Antibiot. (Tokyo) 33: 1054–1062. 7440410 10.7164/antibiotics.33.1054

[bib41] Kawada M. Amemiya M. Ishizuka M. Takeuchi T., 1999 Differential induction of apoptosis in B16 melanoma and EL-4 lymphoma cells by cytostatin and bactobolin. Jpn. J. Cancer Res. 90: 219–225. 10189893 10.1111/j.1349-7006.1999.tb00736.x PMC5926052

[bib42] Knab A. M. Fertala J. Bjornsti M. A., 1993 Mechanisms of camptothecin resistance in yeast DNA topoisomerase I mutants. J. Biol. Chem. 268: 22322–22330. 8226741

[bib43] Kunkel T. A. Erie D. A., 2005 DNA mismatch repair. Annu. Rev. Biochem. 74: 681–710. 15952900 10.1146/annurev.biochem.74.082803.133243

[bib44] Lage H. Dietel M., 1999 Involvement of the DNA mismatch repair system in antineoplastic drug resistance. J. Cancer Res. Clin. Oncol. 125: 156–165. 10235469 10.1007/s004320050258 PMC12199888

[bib45] Lang G. I. Parsons L. Gammie A. E., 2013 Mutation rates, spectra, and genome-wide distribution of spontaneous mutations in mismatch repair deficient yeast. G3 (Bethesda) **3:** 1453–1465. 10.1534/g3.113.006429 PMC3755907 23821616

[bib46] Lerner J., 1989 Choline Transport Specificity in Animal-Cells and Tissues. Comp. Biochem. Physiol. C Pharmacol. Toxicol. Endocrinol. 93: 1–9. 10.1016/0742-8413(89)90002-9 2567220

[bib47] Li Z. Y. Brendel M., 1993 Co-regulation with genes of phospholipid biosynthesis of the CTR/HNM1-encoded choline/nitrogen mustard permease in Saccharomyces cerevisiae. Mol. Gen. Genet. 241: 680–684. 8264542 10.1007/BF00279911

[bib48] Lin X. J. Okuda T. Holzer A. Howell S. B., 2002 The copper transporter CTR1 regulates cisplatin uptake in Saccharomyces cerevisiae. Mol. Pharmacol. 62: 1154–1159. 12391279 10.1124/mol.62.5.1154

[bib49] Lorenz M. C. Heitman J., 1995 TOR mutations confer rapamycin resistance by preventing interaction with FKBP12-rapamycin. J. Biol. Chem. 270: 27531–27537. 7499212 10.1074/jbc.270.46.27531

[bib50] Lum P. Y. Armour C. D. Stepaniants S. B. Cavet G. Wolf M. K., 2004 Discovering modes of action for therapeutic compounds using a genome-wide screen of yeast heterozygotes. Cell 116: 121–137. 14718172 10.1016/s0092-8674(03)01035-3

[bib51] Millard J. T. Raucher S. Hopkins P. B., 1990 Mechlorethamine cross-links deoxyguanosine residues at 5′-GNC sequences in duplex DNA fragments. J. Am. Chem. Soc. 112: 2459–2460.

[bib52] Mondesire W. H. Jian W. G. Zhang H. X. Ensor J. Hung M. C., 2004 Targeting mammalian target of rapamycin synergistically enhances chemotherapy-induced cytotoxicity in breast cancer cells. Clin. Cancer Res. 10: 7031–7042. 15501983 10.1158/1078-0432.CCR-04-0361

[bib53] Nowell P. C., 1976 The clonal evolution of tumor cell populations. Science 194: 23–28. 959840 10.1126/science.959840

[bib54] Parsons A. B. Brost R. L. Ding H. M. Li Z. J. Zhang C. Y., 2004 Integration of chemical-genetic and genetic interaction data links bioactive compounds to cellular target pathways. Nat. Biotechnol. 22: 62–69. 14661025 10.1038/nbt919

[bib55] Parsons A. B. Lopez A. Givoni I. E. Williams D. E. Gray C. A., 2006 Exploring the mode-of-action of bioactive compounds by chemical-genetic profiling in yeast. Cell 126: 611–625. 16901791 10.1016/j.cell.2006.06.040

[bib56] Pommier Y., 2006 Topoisomerase I inhibitors: camptothecins and beyond. Nat. Rev. Cancer 6: 789–802. 16990856 10.1038/nrc1977

[bib57] Rink S. M. Hopkins P. B., 1995 A mechlorethamine-induced DNA interstrand cross-link bends duplex DNA. Biochemistry 34: 1439–1445. 7827092 10.1021/bi00004a039

[bib58] Rosenthal G. A., 1977 The biological effects and mode of action of L-canavanine, a structural analogue of L-arginine. Q. Rev. Biol. 52: 155–178. 331385 10.1086/409853

[bib59] Schenk P. W. Brok M. Boersma A. W. M. Brandsma J. A. Den Dulk H., 2003 Anticancer drug resistance induced by disruption of the Saccharomyces cerevisiae NPR2 gene: a novel component involved in cisplatin- and doxorubicin-provoked cell kill. Mol. Pharmacol. 64: 259–268. 12869630 10.1124/mol.64.2.259

[bib60] Schofield M. J. Hsieh P., 2003 DNA mismatch repair: Molecular mechanisms and biological function. Annu. Rev. Microbiol. 57: 579–608. 14527292 10.1146/annurev.micro.57.030502.090847

[bib61] Sietsma H. Veldman R. J. Kok J. W., 2001 The involvement of sphingolipids in multidrug resistance. J. Membr. Biol. 181: 153–162. 11420602 10.1007/s00232-001-0033-1

[bib62] Stefanko E. Wrobel T., 2010 Mechanisms of resistance to cancer chemotherapy. Adv. Clin. Exp. Med. 19: 5–12.

[bib63] Stubbs M. McSheehy P. M. J. Griffiths J. R. Bashford C. L., 2000 Causes and consequences of tumour acidity and implications for treatment. Mol. Med. Today 6: 15–19. 10637570 10.1016/s1357-4310(99)01615-9

[bib64] Stubbs M. Bashford C. L. Griffiths J. R., 2003 Understanding the tumor metabolic phenotype in the genomic era. Curr. Mol. Med. 3: 49–59. 12558074 10.2174/1566524033361645

[bib65] Surtees J. A. Argueso J. L. Alani E., 2004 Mismatch repair proteins: key regulators of genetic recombination. Cytogenet. Genome Res. 107: 146–159. 15467360 10.1159/000080593

[bib66] Tosato V. Gruning N.-M. Breitenbach M. Arnak R. Ralser M., 2012 Warburg effect and translocation-induced genomic instability: two yeast models for cancer cells. Front. Oncol. 2: 212. 23346549 10.3389/fonc.2012.00212 PMC3548335

[bib67] Veldman R. J. Zerp S. van Blitterswijk W. J. Verheij M., 2004 N-hexanoyl-sphingomyelin potentiates in vitro doxorubicin cytotoxicity by enhancing its cellular influx. Br. J. Cancer 90: 917–925. 14970874 10.1038/sj.bjc.6601581 PMC2410169

[bib68] Vignot S. Faivre S. Aguirre D. Raymond E., 2005 mTOR-targeted therapy of cancer with rapamycin derivatives. Ann. Oncol. 16: 525–537. 15728109 10.1093/annonc/mdi113

[bib69] Weinberg J. Drubin D. G., 2012 Clathrin-mediated endocytosis in budding yeast. Trends Cell Biol. 22: 1–13. 22018597 10.1016/j.tcb.2011.09.001 PMC3253927

[bib70] Westphal C. H. Dipp M. A. Guarente L., 2007 A therapeutic role for sirtuins in diseases of aging? Trends Biochem. Sci. 32: 555–560. 17980602 10.1016/j.tibs.2007.09.008

[bib71] Whelan W. L. Gocke E. Manney T. R., 1979 The CAN1 locus of Saccharomyces cerevisiae: fine-structure analysis and forward mutation rates. Genetics 91: 35–51. 372045 10.1093/genetics/91.1.35 PMC1213930

[bib72] Wu H. I. Brown J. A. Dorie M. J. Lazzeroni L. Brown J. M., 2004 Genome-wide identification of genes conferring resistance to the anticancer agents cisplatin, oxaliplatin, and mitomycin C. Cancer Res. 64: 3940–3948. 15173006 10.1158/0008-5472.CAN-03-3113

[bib73] Zhou B. Gitschier J., 1997 hCTR1: A human gene for copper uptake identified by complementation in yeast. Proc. Natl. Acad. Sci. USA 94: 7481–7486. 9207117 10.1073/pnas.94.14.7481 PMC23847

[bib74] Zimmermann H. F. John G. T. Trauthwein H. Dingerdissen U. Huthmacher K., 2003 Rapid evaluation of oxygen and water permeation through microplate sealing tapes. Biotechnol. Prog. 19: 1061–1063. 12790681 10.1021/bp025774t

