# Supplementary material for: Rapid Identification of Chemoresistance Mechanisms Using Yeast DNA Mismatch Repair Mutants
Source: G3 (Bethesda). 2015 Jul 21;5(9):1925–35. doi: 10.1534/g3.115.020560 (PMC4555229; doi:10.1534/g3.115.020560)
Supplement: Supporting Information [file supp_5_9_1925__index.html]

Rapid Identification of Chemoresistance Mechanisms Using Yeast DNA Mismatch Repair Mutants — Supporting Information 

# Rapid Identification of Chemoresistance Mechanisms Using Yeast DNA Mismatch Repair Mutants

## Supporting Information for Ojini and Gammie, 2015

**Files in this Data Supplement:**

- Supporting Information - Tables S1-S2 and descriptions of Files S1-S6 (PDF, 113 KB)
- Table S1 - Compounds with high resistance rates after prolonged growth. (PDF, 78 KB)
- Table S2 - Compounds with a mismatch repair specific resistance phenotype. (PDF, 77 KB)
- File S1 - Mechanistic Set Raw Data (.xlsx, 187 KB)
- File S2 - Approved Oncology Set Raw Data (.xlsx, 23 KB)
- File S3 - Diversity Set Raw Data (.xlsx, 326 KB)
- File S4 - Natural Products Set Raw Data (.xlsx, 41 KB)
- File S5 - Mutation Event Resistance Set (.xlsx, 294 KB)
- File S6 - Condition of MMR defect Resistance Set (.xlsx, 124 KB)
