# Supplementary material for: Rapid Identification of Chemoresistance Mechanisms Using Yeast DNA Mismatch Repair Mutants
Source: G3 (Bethesda). 2015 Jul 21;5(9):1925–35. doi: 10.1534/g3.115.020560 (PMC4555229; doi:10.1534/g3.115.020560)
Supplement: Supporting Information [file supp_g3.115.020560_020560SI.pdf]

G3 · Genes | Genomes | Genetics

Rapid Identification of Chemoresistance Mechanisms Using Yeast DNA Mismatch Repair Mutants

Irene Ojini and Alison Gammie

Department of Molecular Biology, Princeton University, Princeton, NJ 08544

Corresponding author: Alison Gammie

Office Mailing Address: Department of Molecular Biology, Princeton University, Princeton, NJ 08544-1014; Phone: (609) 258-6380, Fax: (609) 258-1975; E-mail: agammie@princeton.edu

DOI: 10.1534/g3.115.020560

Table S1 Compounds with high resistance rates after prolonged growth

Compounds	*msh2*Δ Lag Phase (hours)	Notes
NSC 1011	41	1st NIH Screen
Celastrol	24	1st NIH Screen
Actinomycin D	36	2nd NIH Screen
Camptothecin	39	2nd NIH Screen
Daunorubicin	29	2nd NIH Screen
Mitoxantrone	>24	3rd Screen
Exemestane	>24	3rd Screen
Mechlorethamine	>24	3rd Screen
Hexestrol	>24	3rd Screen
Doxorubicin	>24	3rd Screen
Cinnarizine	>24	3rd Screen
Doxycycline	>24	3rd Screen
Aphidicolin	>24	3rd Screen
YC-1	>24	3rd Screen
Myriocin	>24	3rd Screen
Cerulenin	>24	3rd Screen
Perillic Acid	>24	3rd Screen
Brefeldin A	>24	3rd Screen
MST-312	>24	3rd Screen

Table S2 Compounds with a mismatch repair specific resistance phenotype

Compounds	*msh2*Δ Lag Phase (hours)	Screen
Thiallipticine	17	1st NIH Screen
NSC 116339	18	1st NIH Screen
Confertifoline	13	2nd NIH Screen
Etoposide	<24	3rd Screen
Dacarbazine	<24	3rd Screen
Fluorouracil	<24	3rd Screen
Piperine	<24	3rd Screen
Formestane	<24	3rd Screen
Estramustine	<24	3rd Screen

Files S1-S6

Available for download as Excel files at www.g3journal.org/lookup/suppl/doi:10.1534/g3.115.020560/-/DC1

File S1 Mechanistic Set Raw Data

File S2 Approved Oncology Set Raw Data

File S3 Diversity Set Raw Data

File S4 Natural Products Set Raw Data

File S5 Mutation Event Resistance Set

File S6 Condition of MMR defect Resistance Set
