# Supplementary material for: Rapid Identification of Chemoresistance Mechanisms Using Yeast DNA Mismatch Repair Mutants
Source: G3 (Bethesda). 2015 Jul 21;5(9):1925–35. doi: 10.1534/g3.115.020560 (PMC4555229; doi:10.1534/g3.115.020560)
Supplement: Supporting Information [file supp_g3.115.020560_TableS1.pdf]

Table S1 Compounds with high resistance rates after prolonged growth

**Compounds**	***msh2*Δ Lag Phase (hours)**	**Notes**
NSC 1011	41	1st NIH Screen
Celastrol	24	1st NIH Screen
Actinomycin D	36	2nd NIH Screen
Camptothecin	39	2nd NIH Screen
Daunorubicin	29	2nd NIH Screen
Mitoxantrone	>24	3rd Screen
Exemestane	>24	3rd Screen
Mechlorethamine	>24	3rd Screen
Hexestrol	>24	3rd Screen
Doxorubicin	>24	3rd Screen
Cinnarizine	>24	3rd Screen
Doxycycline	>24	3rd Screen
Aphidicolin	>24	3rd Screen
YC-1	>24	3rd Screen
Myriocin	>24	3rd Screen
Cerulenin	>24	3rd Screen
Perillic Acid	>24	3rd Screen
Brefeldin A	>24	3rd Screen
MST-312	>24	3rd Screen
