# Supplementary material for: Rapid Identification of Chemoresistance Mechanisms Using Yeast DNA Mismatch Repair Mutants
Source: G3 (Bethesda). 2015 Jul 21;5(9):1925–35. doi: 10.1534/g3.115.020560 (PMC4555229; doi:10.1534/g3.115.020560)
Supplement: Supporting Information [file supp_g3.115.020560_TableS2.pdf]

Table S2   Compounds with a mismatch repair specific resistance phenotype

**Compounds**	***msh2*Δ Lag Phase (hours)**	**Screen**
Thiallipticine	17	1st NIH Screen
NSC 116339	18	1st NIH Screen
Confertifoline	13	2nd NIH Screen
Etoposide	<24	3rd Screen
Dacarbazine	<24	3rd Screen
Fluorouracil	<24	3rd Screen
Piperine	<24	3rd Screen
Formestane	<24	3rd Screen
Estramustine	<24	3rd Screen
